# Association Between Task Difficulty Regulation in Game-Based Digital Therapeutics and Attention-Deficit/Hyperactivity Disorder Symptoms in Children: Secondary Analysis of a Randomized Controlled Trial

**DOI:** 10.2196/83932

**Published:** 2026-06-11

**Authors:** Jun-Su Kim, Seung-Jae Kim, Gilnam Ryu, Su-Jin Jun, Jin-Yeop Park, Mun-Ju Kim, Sang Sun Han, So-Hyeon Yoo, Byeong-II Kim, Hyun-ju Lee, Jeong-Heon Song, Hyang-Sook Hoe

**Affiliations:** 1 AI-Based Neurodevelopmental Diseases Digital Therapeutics Group, Korea Brain Research Institute (KBRI), 61, Cheomdan-ro, Daegu, 41062, Republic of Korea, 82 53-980-8310; 2 Department of Neural Development and Disease, Korea Brain Research Institute (KBRI), Daegu, Republic of Korea; 3 Woorisoft Inc, Daegu, Republic of Korea; 4 Department of Brain and Cognitive Sciences, Daegu Gyeongbuk Institute of Science & Technology, Daegu, Republic of Korea

**Keywords:** attention-deficit/hyperactivity disorder, cognitive training, digital therapeutics, game-based digital content, task performance, task difficulty levels

## Abstract

**Background:**

Recent advances in digital therapeutics (DTx) have led to the development of game-based, home-delivered interventions for children with attention-deficit/hyperactivity disorder (ADHD). Prior studies have demonstrated the feasibility and clinical potential of DTx for reducing ADHD symptoms but have primarily evaluated usage metrics, including training frequency and duration, which do not capture in-game performance or learning processes during training. In addition, the association between task difficulty regulation during DTx training and ADHD symptom improvement in children has not been well studied.

**Objective:**

This study aimed to examine whether task difficulty regulation during DTx training is associated with ADHD symptom improvement in children and to evaluate the clinical relevance of difficulty-related behavioral measures as indicators of treatment response.

**Methods:**

In this study, a secondary post hoc analysis of data from 35 children aged 6 to 13 years with ADHD who participated in a randomized controlled trial was performed. The participants had completed a 4-week training program comprising 5 digital cognitive tasks from the DTx Neuro-World. During training, task difficulty was automatically adjusted in real time according to participants’ response accuracy to maintain an appropriate challenge level. Training difficulty was quantified by 3 indices: average task difficulty, task difficulty variability, and task difficulty slope. Associations between difficulty indicators and ADHD symptom changes were examined using regression analyses and group comparisons.

**Results:**

We found that lower average task difficulty during tasks 1, 2, and 4 of Neuro-World was associated with improvements in ADHD symptoms, as assessed by the total Korean-ADHD Rating Scale (K-ARS) score. In addition, the average task difficulty of tasks 1 to 4 was related to improvements in hyperactivity/impulsivity symptoms of ADHD, as assessed by the K-ARS hyperactivity or impulsivity subscore, but not to improvements in inattention symptoms. Moreover, lower variability of task difficulty in task 5 improved ADHD hyperactivity/impulsivity symptoms, as assessed by K-ARS, but did not affect Korean-Child Behavior Checklist scores. Finally, we found that a negative task difficulty slope was associated with the alleviation of ADHD hyperactivity or impulsivity. These findings should be interpreted with caution, given the exploratory nature of the study and the absence of formal correction for multiple comparisons.

**Conclusions:**

As an exploratory investigation, our study provides a novel perspective by demonstrating that individualized and stable regulation of task difficulty, rather than the mere attainment of higher difficulty levels, is associated with ADHD symptom improvement. These findings indicate that dynamic difficulty trajectories derived from in-game behavioral data are clinically relevant, going beyond the conventional focus in the literature on usage metrics or overall training intensity. Our results support the role of individualized difficulty trajectories as potential digital biomarkers of clinical response. This approach has important implications for the design of DTx that incorporate personalized and adaptive difficulty regulation to enhance clinical effectiveness and scalability.

## Introduction

Attention-deficit/hyperactivity disorder (ADHD) is a common neurodevelopmental disorder that affects approximately 5% of children and adolescents worldwide [1]. Children with ADHD often experience persistent difficulties with attention, impulse control, and activity level, which can interfere with academic performance, classroom behavior, and peer relationships [2]. These difficulties frequently persist into adolescence and adulthood, affecting daily functioning, emotional development, and long-term quality of life [3,4]. Although stimulant medication and behavioral therapy are well-established first-line treatments for ADHD, their effectiveness varies across individuals [5]. Many children discontinue medication because of adverse effects, such as appetite suppression or sleep disturbances [6,7], and access to behavioral therapy is often limited by financial, geographic, or scheduling constraints [8]. Real-world effectiveness may also be influenced by family involvement and environmental support [9,10]. These limitations emphasize the importance of developing treatment approaches that are accessible, sustainable, and feasible in real-world settings. Interventions that actively engage children, reduce treatment burden, and can be integrated into daily routines offer a practical approach to this challenge.

Digital therapeutics (DTx) have emerged as an innovative approach to supplement or extend traditional ADHD treatments through software-based therapeutic programs [11,12]. These interventions use structured, evidence-informed tasks to target cognitive processes such as attention, response control, and working memory [13,14]. A key strength of DTx is their ability to deliver therapeutic content in everyday environments, which allows children to engage in treatment at home, within their daily routines [15-17]. Game-based formats support motivation and sustained engagement in repeated practice without the fatigue or disengagement often associated with traditional cognitive exercises, making them particularly suitable for pediatric populations [18,19]. For example, EndeavorRx, the first Food and Drug Administration–authorized prescription DTx for pediatric ADHD, has been shown to improve attention performance among children aged 8 to 12 years, including those who discontinued medication [11]. Virtual reality–based digital programs have similarly been shown to reduce impulsive or aggressive behavior and improve social responsiveness [20]. Collectively, these findings suggest that DTx can produce clinically meaningful benefits by combining therapeutic mechanisms with engaging and adaptive delivery formats [21]. Continued advances in DTx increasingly emphasize the development of personalized and adaptive DTx to address symptom heterogeneity in children with ADHD.

Cognitive training using DTx also generates performance-level behavioral data, which have been underutilized in DTx research. Most previous studies have relied on usage-based indicators such as total training time, number of sessions completed, or session duration [22-24]. These values reflect engagement frequency but provide limited insight into cognitive performance quality or learning processes during training. Game-based DTx platforms commonly use adaptive algorithms that adjust task difficulty in real time based on an individual’s performance [25,26]. Although task difficulty adaptation is a central feature of many DTx systems, its clinical relevance has rarely been examined directly. Studies of task-difficulty dynamics within web-based cognitive training programs for stroke rehabilitation have suggested that difficulty regulation patterns convey clinically meaningful information, but comparable research in ADHD populations remains scarce [27]. Specifically, it remains unclear how training under task difficulty levels matched to a child’s capacity and ability to maintain or regulate those levels across repeated sessions of DTx is related to ADHD symptom improvement. Clarifying this relationship between performance and ADHD symptom improvement or impairment is essential for advancing both the understanding and development of DTx.

Based on the literature and our recent findings in children with ADHD who used the DTx Neuro-World [28-31], we hypothesized that stable and individualized regulation of task difficulty improves ADHD symptoms. Our hypothesis departs from previous approaches focused on increasing absolute difficulty levels. To test this hypothesis, this study examined associations between quantified difficulty patterns and changes in attention, hyperactivity/impulsivity, and overall behavioral functioning in children with ADHD.

## Methods

### Recruitment

This study enrolled pediatric patients aged 6 to 13 years who had been clinically diagnosed with ADHD at the outpatient pediatric psychiatry clinic of Keimyung University Dongsan Hospital in the Republic of Korea. Participants were selected using clinic-based convenience sampling during routine outpatient visits. In the first phase, 34 children were recruited from December 2021 to December 2022; in the next phase, an additional 34 children were enrolled from December 2022 to March 2023. Data collection occurred concurrently with each recruitment phase.

### Recruitment Sampling Procedures

Recruitment was conducted using a convenience sampling strategy. Eligible children and their legal guardians were informed about the study by clinicians or trained research staff during routine clinic visits, and participants who met the inclusion criteria were consecutively enrolled. As part of the original trial design, all enrolled participants were randomly assigned to either the intervention group (DTx) or the control group (non-DTx).

### Inclusion and Exclusion

Children with comorbid neurological or psychiatric conditions other than ADHD, including posttraumatic stress disorder, psychosis, severe obsessive-compulsive disorder, or severe depression, were excluded. Participants with physical limitations that could interfere with the use of the DTx program (eg, deformities of the hands or arms or the use of prosthetic limbs) were also excluded. In addition, 4 children declined participation.

### Participant Characteristics

The original randomized controlled trial enrolled a total of 64 children, who were randomly assigned to either the control group (n=29) or the DTx intervention group (n=35). Building on the primary study [28-30], this study represents a secondary post hoc analysis focused exclusively on the participants who completed the DTx intervention. Accordingly, only the data from the 35 children assigned to the intervention group were analyzed in this study because the primary objective was to examine associations between in-game behavioral metrics and ADHD symptom changes derived from DTx training-session data, rather than to compare intervention efficacy between the control and DTx intervention groups. All participants were Korean and were recruited from a single clinical institution. Socioeconomic status and the education level of the legal guardian were not collected.

### Sample Size, Power, and Precision

The target sample size was determined based on feasibility and previous literature, with an anticipated attrition rate of approximately 10%, and a total of 64 participants were enrolled (control group: 29; intervention group: 35). An a priori power analysis conducted using G*Power (version 3.1.9.7) indicated that a minimum of 24 participants was required to detect a medium-to-large effect size (*d*z=0.6; *α*=.05; power=0.80) in paired comparisons, confirming that the target sample size was achieved. For this secondary analysis, 35 participants in the intervention group who completed the training were included. Given the negligible attrition, the final sample size remained consistent with the original assumptions. A post hoc power analysis for the regression models, assuming a medium-to-large effect size (*f*²=0.35; *α*=.05; number of predictors=2), indicated that a minimum of 31 participants was required. The achieved sample size of 35 yielded an observed power of 0.80, confirming that the analysis was sufficiently powered. No interim analyses or formal stopping rules were applied. As this is an exploratory secondary analysis, the findings should be interpreted with caution, particularly regarding the precision of parameter estimates.

### Ethical Considerations

Phase 1 of the study was approved and registered by the institutional review board of Keimyung University Dongsan Hospital (number 2021-10-080), and phase 2 was approved and registered with the Korea Clinical Research Information Service (number KCT0009326). Prior to enrollment, written informed consent was obtained from the legal guardians of all participants, and the experimental procedures were explained to the participants to obtain their assent. The consent form explicitly indicated that anonymized data could be used for clinical outcome reporting as well as for secondary analyses and future research without requiring additional consent. Participants received modest compensation of approximately US $200, equivalent to approximately 270,000 Korean won, upon the completion of the study.

All study data were deidentified prior to analysis, and each participant was assigned a unique study identification code; no direct personal identifiers were retained in the analytical dataset. Data handling procedures complied with applicable institutional and regulatory guidelines to ensure the privacy and confidentiality of all participants. No personally identifiable information is included in this study or its supplementary materials, and access to the deidentified data was restricted to authorized research personnel.

### Data Collection

**Figure 1.**
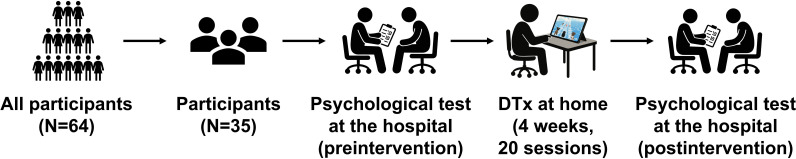
Overview of the experimental procedure for participants in the digital therapeutics (DTx) intervention group. This figure illustrates the overall study flow, including participant enrollment, baseline assessment, the 4-week home-based Neuro-World digital cognitive training intervention, and the postintervention assessment conducted within 1 week after the completion of all DTx training sessions. The original randomized controlled trial enrolled 64 children with attention-deficit/hyperactivity disorder (ADHD); however, only the 35 participants assigned to the DTx intervention group were included in the post hoc analysis for this study.

To minimize potential confounding effects on treatment response, participants were instructed to maintain their usual daily routines and to continue prescribed medications without adjustment. After informed consent was obtained, all participants completed baseline assessments using the Korean-ADHD Rating Scale (K-ARS) and the Korean-Child Behavior Checklist (K-CBCL) to assess ADHD symptoms and emotional and behavioral functioning. Following the baseline assessment, participants in the intervention group completed a home-based digital cognitive training program. The DTx intervention consisted of 20 training sessions delivered over 4 weeks, with 5 sessions per week (Figure 1).

Home-based training allowed participants to complete sessions in a familiar environment while maintaining flexibility in scheduling. To ensure that task performance reflected genuine cognitive engagement, caregivers were instructed to provide a quiet and independent setting during each session, thereby minimizing environmental distractions. Within 1 week after the completion of all training sessions, participants returned to the clinic for postintervention assessment, during which the standardized measures administered at baseline were repeated.

### Instrumentation: K-ARS and K-CBCL

The K-ARS is a widely used standardized instrument for assessing ADHD-related symptoms in children and adolescents. It was adapted for Korean populations from the ADHD Rating Scale-IV, the reliability and validity of which had previously been established [32]. The K-ARS is a reliable and clinically useful tool for evaluating, screening, and monitoring ADHD symptomatology in Korean children. It consists of 18 behavior-rating items reflecting the *DSM* (*Diagnostic and Statistical Manual of Mental Disorders*)-based ADHD symptom domains, including 9 items assessing inattention and 9 items assessing hyperactivity-impulsivity [33,34]. Each item is rated on a 4-point scale, with higher scores indicating greater symptom severity [34]. The instrument is typically completed by caregivers or teachers, and both forms are recognized for their high validity and reliability in assessing ADHD-related behaviors [19,33].

The K-CBCL is a culturally validated adaptation of the widely used Child Behavior Checklist, which was originally developed by Achenbach and Rescorla and translated and standardized in Korea by Oh and Kim [35,36]. The Child Behavior Checklist was subsequently adapted for Korean populations to create the K-CBCL, which consists of 121 parent-reported items that assess children’s behavioral and emotional problems [37]. As a standardized instrument for caregivers of children aged 6 to 18 years, the K-CBCL evaluates adaptive functioning and problematic behaviors observed during the previous 6 months [35]. The K-CBCL includes several empirically derived syndrome subscales, such as anxious/depressed, withdrawn/depressed, somatic complaints, social problems, thought problems, attention problems, rule-breaking behavior, and aggressive behavior [38]. These subscales are further organized into internalizing problems, which comprise anxious/depressed, withdrawn/depressed, and somatic complaints, and externalizing problems, which include rule-breaking behavior and aggressive behavior as the primary domains [39]. Collectively, the subscales of the K-CBCL provide a comprehensive assessment of emotional, behavioral, and social functioning, and the K-CBCL serves as a reliable and widely used measure for screening and monitoring psychological symptoms in Korean children.

The K-ARS provides a focused evaluation of inattention and hyperactivity or impulsivity in children with ADHD, whereas the K-CBCL captures a wide spectrum of internalizing and externalizing problems as well as social and behavioral adjustment. Together, the K-ARS and K-CBCL give complementary perspectives on ADHD-specific symptoms and broader behavioral functioning, enabling a multidimensional assessment of ADHD symptom change over time.

### Instrumentation: Cognitive Training Program

**Figure 2.**
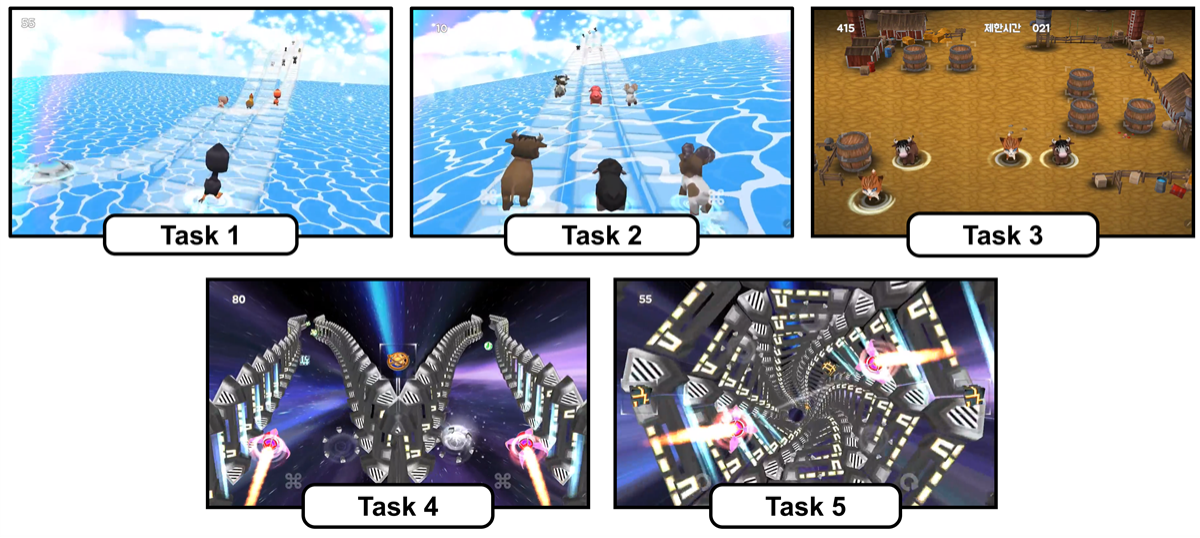
The cognitive digital tasks of the game-based digital therapeutics (DTx) software Neuro-World: (1) identifying shared features of animal figures under dynamically changing visual conditions, (2) rearranging sequences of animals to match target patterns, (3) remembering and recalling animal locations to form matching pairs, (4) independently controlling 2 spaceships to collect targets while avoiding obstacles, and (5) coordinating dual spaceship control to perform both actions simultaneously.

Neuro-World is a game-based DTx software developed by Woorisoft Co, Ltd, to enhance cognitive function in children with ADHD (Figure 2). The DTx contents include five cognitive tasks, each lasting approximately 5 minutes: (1) identifying shared features among animal figures under continuously changing visual conditions, (2) rearranging sequences of animals to match target patterns, (3) memorizing and recalling animal positions to find matching pairs, (4) independently controlling left and right spaceships to collect target items while avoiding obstacles, and (5) coordinating dual spaceship control to collect targets and avoid obstacles simultaneously. A CONSORT-eHEALTH (Consolidated Standards of Reporting Trials of Electronic and Mobile Health Applications and Online Telehealth) checklist describing the Neuro-World intervention and study procedures was completed and submitted separately for editorial review [40] (Checklist 1). We previously demonstrated the effectiveness of Neuro-World in improving cognitive processing speed, working memory, decision-making, and core symptoms of ADHD, and ongoing research is examining its scalability and long-term effects [28-30]. In this study, we further investigated the effects of task difficulty regulation in Neuro-World on ADHD symptom improvement or impairment in the 35 children with ADHD in the intervention group.

### Data Sources

Task difficulty in Neuro-World was continuously modified in real time according to the participants’ digital task performance to maintain an optimal level of task engagement. Difficulty levels were set to range from 1 (lowest) to 20 (highest). Task difficulty was increased after reaching task-specific predefined thresholds of consecutive correct responses and decreased following consecutive incorrect responses. Depending on the DTx task, greater difficulty was achieved by increasing either the task speed or its cognitive load, such as processing or retaining information. To determine whether task difficulty dynamics differentially affected ADHD symptom improvement, we used 3 indicators derived from the task difficulty-adjustment system: the average task difficulty, task difficulty variability, and task difficulty slope (Table 1). These 3 indicators represent distinct aspects of how the DTx task difficulty regulates the DTx training process and served as the main behavioral variables in the analysis.

**Table 1.** Operational definitions and detailed descriptions of task difficulty indicators derived from the Neuro-World digital therapeutics (DTx).

Indicator	Definition	Interpretation
Average task difficulty	The mean task difficulty level across 20 training sessions for each of the 5 tasks.	The overall individual difficulty level during DTx training for each task. Higher values indicate that more difficult levels were performed.
Task difficulty variability	The SD of task difficulty across 20 training sessions for each of the 5 tasks.	Individual variability in task difficulty during DTx training for each task. Higher values indicate greater variability, whereas lower values indicate more consistent performance.
Task difficulty slope	The linear trend of task difficulty across 20 training sessions for each of the 5 tasks, estimated using a regression model.	The rate of difficulty changes over time (β₁). Positive values indicate increasing task difficulty during training, whereas negative values indicate decreasing task difficulty.

### Data Analysis

Data analyses were conducted using regression models and independent-samples 2-tailed *t* tests with data from the 35 participants who received the DTx intervention. Separate regression models were constructed for average task difficulty and task difficulty variability, with postintervention psychological outcomes entered as dependent variables and baseline scores included as covariates to control for initial symptom severity. Because the game-based indicators were derived from multiple tasks with similar data structures, strong intercorrelations were observed. Including highly correlated indicators within a single model could introduce multicollinearity and compromise interpretability [41]. Accordingly, each indicator for the 35 participants who received the DTx intervention was analyzed using a separate regression model. Task difficulty slope represents a regression-derived parameter (β) based on task difficulty over time and was therefore not included as a continuous predictor in an additional regression model. Instead, task difficulty slope values were categorized into positive and negative groups, and independent-samples 2-tailed *t* tests were conducted to compare psychological outcomes between the groups. This approach allowed an examination of whether the direction of task difficulty change was associated with differences in clinical measures. No missing data were identified for psychological or task-derived variables in the DTx intervention group (n=35); therefore, all participants were included in the analyses.

## Results

Only the data from the 35 children assigned to the intervention group were analyzed in this study because the primary objective was to examine associations between in-game behavioral metrics and ADHD symptom changes (Table 2). Among these 35 children, 29 (82.9%) were male and 6 (17.1%) were female, with a mean age of 10.17 (SD 2.43) years. The baseline and postintervention score distributions of K-ARS and K-CBCL scores for 35 subjects are shown in Table 3.

**Table 2.** Demographic characteristics of the digital therapeutics (DTx) intervention group.

Demographic characteristics	Values
Gender, n (%)	
Male	29 (82.9)
Female	6 (17.1)
Total	35 (100)
Age (y), mean (SD; range)	10.17 (2.43; 6‐13)

**Table 3.** Korean-Child Behavior Checklist (K-CBCL) and Korean-ADHD Rating Scale (K-ARS) scores from baseline to postintervention in the digital therapeutics (DTx) intervention group.

Scales	Baseline, mean (SD)	Postintervention, mean (SD)
K-CBCL (N=35)		
Total score	67.37 (10.46)	57.29 (9.53)
Internalizing problems	63.51 (10.29)	54.60 (9.92)
Externalizing problems	64.51 (11.44)	56.17 (8.74)
K-ARS (N=35)		
Total score	19.57 (9.34)	12.03 (7.59)
Inattention	10.09 (5.16)	6.97 (3.99)
Hyperactivity/impulsivity	9.49 (5.71)	5.06 (4.28)

### Associations Between Average Task Difficulty and ADHD Symptoms

Neuro-World was previously shown to attenuate ADHD symptoms in the DTx intervention-treated participants [28-30] (Table 3). Here, we further examined the effects of digital task difficulty on ADHD symptom improvement in the DTx intervention group. Task difficulty was quantified using 3 indicators: average task difficulty, task difficulty variability, and task difficulty slope.

We first evaluated whether the average task difficulties of tasks 1 to 5 modulated ADHD inattention and hyperactivity/impulsivity as assessed by the K-ARS (to assess inattention and hyperactivity/impulsivity in ADHD) and the K-CBCL (to measure internalizing and externalizing symptoms). The results of multiple regression analyses showed that the average task difficulties of tasks 1, 2, and 4 were significantly associated with ADHD symptoms as measured by the total K-ARS score, whereas none of the tasks were significantly associated with K-CBCL scores (Table 4, Multimedia Appendix 1). Among K-ARS domains, the average task difficulties of tasks 1 to 4 were associated with K-ARS hyperactivity/impulsivity scores with regression coefficients of 0.353, 0.337, 1.927, and 0.382, respectively, whereas effects on K-ARS inattention scores were not observed (Table 4). Positive regression coefficients indicate that higher average task difficulty was associated with higher postintervention symptom scores, reflecting a smaller degree of symptom improvement. Taken together, these findings indicate that training at lower average task difficulty was associated with improvements in specific ADHD symptoms, namely hyperactivity/impulsivity.

**Table 4.** Multiple regression analysis of average task difficulty and Korean-ADHD Rating Scale (K-ARS) scores in digital therapeutics (DTx) intervention participants.

Outcome measure/covariate (baseline), and content task		Coefficient, β (95% CI)	*t* test (*df*)	*P* value
K-ARS-to^a^ (postintervention)/K-ARS-to (baseline)				
Task 1		0.617 (0.023 to 1.210)	2.117 (32)	.04
Task 2		0.617 (0.051 to 1.183)	2.221 (32)	.03
Task 3		2.313 (−1.229 to 5.856)	1.330 (32)	.19
Task 4		0.629 (0.052 to 1.205)	2.222 (32)	.03
Task 5		0.368 (−0.295 to 1.031)	1.130 (32)	.27
K-ARS-in^b^ (postintervention)/K-ARS-in (baseline)				
Task 1		0.259 (−0.053 to 0.571)	1.690 (32)	.10
Task 2		0.281 (−0.015 to 0.577)	1.930 (32)	.06
Task 3		0.409 (−1.446 to 2.264)	0.449 (32)	.66
Task 4		0.234 (−0.072 to 0.539)	1.558 (32)	.13
Task 5		0.069 (−0.275 to 0.414)	0.410 (32)	.68
K-ARS-hi^c^ (postintervention)/K-ARS-hi (baseline)				
Task 1		0.353 (0.041 to 0.665)	2.306 (32)	.03
Task 2		0.337 (0.036 to 0.638)	2.282 (32)	.03
Task 3		1.927 (0.092 to 3.762)	2.138 (32)	.04
Task 4		0.382 (0.085 to 0.680)	2.616 (32)	.01
Task 5		0.289 (−0.055 to 0.634)	1.713 (32)	.10

a K-ARS-to: total score.

b K-ARS-in: inattention score.

c K-ARS-hi: hyperactivity or impulsivity score.

### Associations Between Task Difficulty Variability and Changes in ADHD Symptoms

Given that we observed a positive correlation between average task difficulty and ADHD hyperactivity/impulsivity, we next performed multiple regression analyses to examine the associations between task difficulty variability and K-ARS and K-CBCL scores.

We found that the task difficulty variability of task 5 was positively associated with postintervention ADHD hyperactivity/impulsivity, as measured by the K-ARS hyperactivity/impulsivity subscore (*β*=0.721), but not with the K-ARS total score or the K-ARS inattention subscore (Table 5). However, the task difficulty variability of tasks 1 to 4 did not affect the total K-ARS score or the K-ARS inattention and hyperactivity/impulsivity subscores (Table 5). Moreover, the task difficulty variability of tasks 1 to 5 was not associated with the K-CBCL subscale scores (Multimedia Appendix 1). The positive regression coefficients indicate that higher variability in task 5 difficulty was associated with higher postintervention hyperactivity/impulsivity scores, reflecting a smaller degree of symptom improvement. These data suggest that children who trained with more consistent task difficulty levels tended to experience greater alleviation of ADHD hyperactivity/impulsivity symptoms. However, this finding should be interpreted with caution, as the *P* value (.05) was close to the significance threshold.

**Table 5.** Multiple regression analysis of task difficulty variability and Korean-ADHD Rating Scale (K-ARS) scores in digital therapeutics (DTx) intervention participants.

Outcome measure/covariate (baseline), and content task		Coefficient, β (95% CI)	*t* test (*df*)	*P* value
K-ARS-to^a^ (postintervention)/K-ARS-to (baseline)				
Task 1		1.090 (−1.025 to 3.206)	1.050 (32)	.30
Task 2		1.315 (−0.332 to 2.961)	1.627 (32)	.11
Task 3		2.021 (−2.016 to 6.057)	1.020 (32)	.32
Task 4		1.179 (−0.136 to 2.493)	1.826 (32)	.08
Task 5		1.001 (−0.369 to 2.372)	1.488 (32)	.15
K-ARS-in^b^ (postintervention)/K-ARS-in (baseline)				
Task 1		0.211 (−0.899 to 1.320)	0.387 (32)	.70
Task 2		0.473 (−0.401 to 1.348)	1.103 (32)	.28
Task 3		0.232 (−1.846 to 2.310)	0.227 (32)	.82
Task 4		0.475 (−0.207 to 1.157)	1.419 (32)	.17
Task 5		0.256 (−0.461 to 0.973)	0.726 (32)	.47
K-ARS-hi^c^ (postintervention)/K-ARS-hi (baseline)				
Task 1		0.821 (−0.264 to 1.906)	1.542 (32)	.13
Task 2		0.834 (−0.024 to 1.692)	1.980 (32)	.06
Task 3		1.819 (−0.295 to 3.933)	1.753 (32)	.09
Task 4		0.674 (−0.017 to 1.364)	1.988 (32)	.06
Task 5		0.721 (0.014 to 1.427)	2.078 (32)	.046

a K-ARS-to: total score.

b K-ARS-in: inattention score.

c K-ARS-hi: hyperactivity/impulsivity score.

### Comparison of ADHD Symptom Changes as a Function of Negative or Positive Task Difficulty Slope

**Figure 3.**
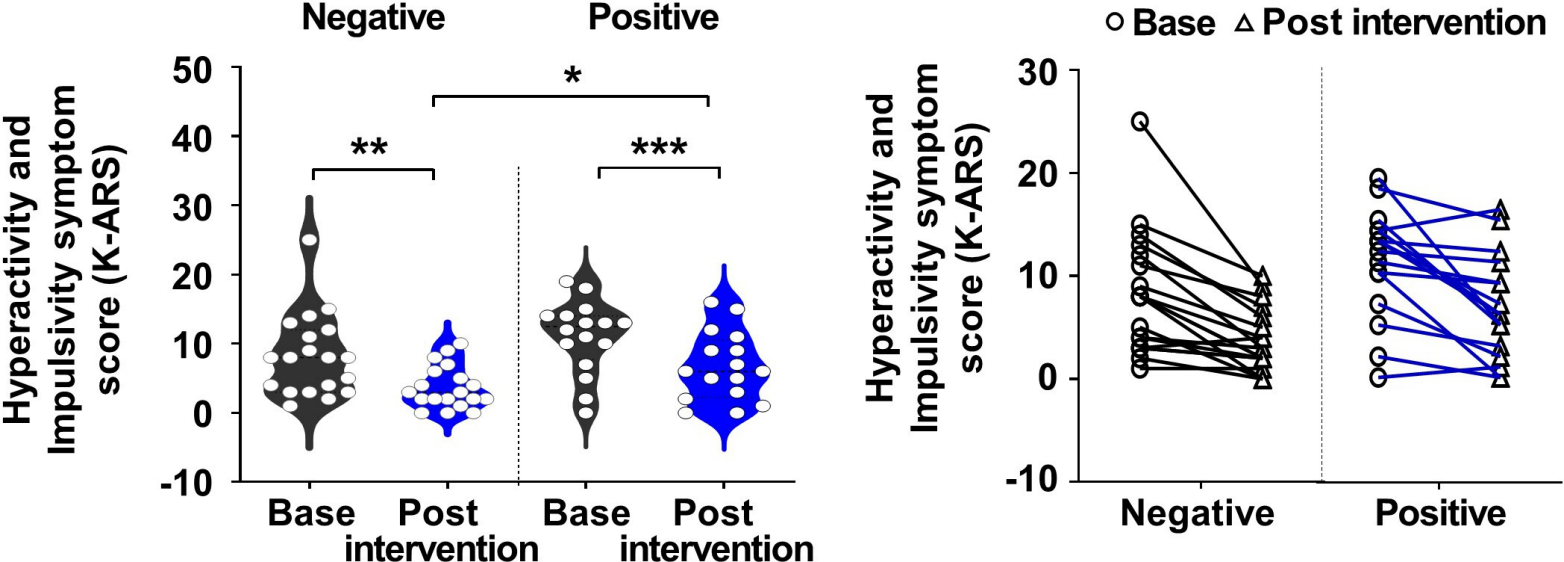
Associations between digital therapeutics (DTx) task difficulty slopes and Korean-ADHD Rating Scale (K-ARS) hyperactivity/impulsivity scores. (Left) Results of paired-sample 2-tailed *t* tests comparing symptom changes between participants with negative (n=19) versus positive (n=16) task difficulty slopes. (Right) Corresponding baseline and posttraining psychological assessment scores for each task difficulty slope group (*P<.05, **P<.01, ***P<.001).

To determine the effects of task difficulty slope on ADHD symptoms, the participants who received the DTx intervention were classified into positive and negative task difficulty slope groups, and independent-samples 2-tailed *t* tests were conducted. Baseline K-ARS hyperactivity/impulsivity scores did not differ between the negative slope group (mean 8.21, SD 5.89) and the positive slope group for any task (mean 11.00, SD 5.27; *P*>.05). Only the postintervention task difficulty slope of task 1 was associated with a between-group difference in ADHD hyperactivity/impulsivity, whereas the negative and positive task difficulty slope groups did not differ for tasks 2 to 5 (Table 6). Specifically, for task 1, the postintervention K-ARS hyperactivity/impulsivity scores differed significantly between the negative task difficulty slope group (mean 3.68, SD 3.06) and the positive task difficulty slope group (mean 6.69, SD 5.02; *P*=.04; Figure 3). However, significant differences in postintervention K-CBCL scores compared with baseline were not observed in the negative or positive task difficulty slope groups for any of the tasks (Multimedia Appendix 1).

**Table 6.** Independent-samples 2-tailed *t* test results comparing baseline and postintervention Korean-ADHD Rating Scale (K-ARS) outcomes between positive and negative task difficulty slope groups.

Performance feature, psychological variable, and session		Participants, n (%)		Mean (SD)		95% CI	*t* test (*df*)	*P* value
		A^a^	B^b^	A^a^	B^b^			
Task 1								
K-ARS-to^c^								
Baseline		19 (54.3)	16 (45.7)	19.05 (10.04)	20.19 (8.72)	−5.080 to 7.349	0.354 (33)	.73
Postintervention		19 (54.3)	16 (45.7)	10.47 (5.98)	13.88 (9.00)	−1.764 to 8.567	1.336 (33)	.19
K-ARS-in^d^								
Baseline		19 (54.3)	16 (45.7)	10.84 (5.69)	9.19 (4.48)	−5.024 to 1.715	−0.943 (33)	.35
Postintervention		19 (54.3)	16 (45.7)	6.79 (3.58)	7.19 (4.53)	−2.347 to 3.143	0.290 (33)	.77
K-ARS-hi^e^								
Baseline		19 (54.3)	16 (45.7)	8.21 (5.89)	11.00 (5.27)	−0.909 to 6.488	1.464 (33)	.15
Postintervention		19 (54.3)	16 (45.7)	3.68 (3.06)	6.69 (5.02)	0.187 to 5.819	2.177 (33)	.04
Task 2								
K-ARS-to								
Baseline		10 (28.6)	25 (71.4)	17.00 (10.31)	20.60 (8.93)	−3.685 to 10.885	1.031 (33)	.31
Postintervention		10 (28.6)	25 (71.4)	10.30 (6.98)	12.72 (7.85)	−2.888 to 7.728	0.849 (33)	.40
K-ARS-in								
Baseline		10 (28.6)	25 (71.4)	9.50 (5.97)	10.32 (4.92)	−3.352 to 4.992	0.419 (33)	.68
Postintervention		10 (28.6)	25 (71.4)	6.80 (4.18)	7.04 (3.99)	−2.790 to 3.270	0.158 (33)	.88
K-ARS-hi								
Baseline		10 (28.6)	25 (71.4)	7.50 (5.52)	10.28 (5.70)	−1.307 to 6.867	1.315 (33)	.20
Postintervention		10 (28.6)	25 (71.4)	3.50 (3.27)	5.68 (4.53)	−0.518 to 4.878	1.378 (33)	.18
Task 3								
K-ARS-to								
Baseline		19 (54.3)	16 (45.7)	20.42 (9.97)	18.56 (8.74)	−8.058 to 4.341	−0.581 (33)	.57
Postintervention		19 (54.3)	16 (45.7)	11.95 (7.07)	12.13 (8.40)	−5.023 to 5.379	0.068 (33)	.95
K-ARS-in								
Baseline		19 (54.3)	16 (45.7)	11.26 (5.54)	8.69 (4.45)	−5.886 to 0.735	−1.496 (33)	.14
Postintervention		19 (54.3)	16 (45.7)	7.68 (3.93)	6.13 (4.01)	−4.204 to 1.085	−1.158 (33)	.26
K-ARS-hi								
Baseline		19 (54.3)	16 (45.7)	9.16 (5.85)	9.88 (5.70)	−3.121 to 4.555	0.365 (33)	.72
Postintervention		19 (54.3)	16 (45.7)	4.26 (3.69)	6.00 (4.84)	−1.160 to 4.634	1.203 (33)	.24
Task 4								
K-ARS-to								
Baseline		7 (20)	28 (80)	22.00 (14.00)	18.96 (8.02)	−13.824 to 7.753	−0.765 (33)	.45
Postintervention		7 (20)	28 (80)	11.00 (8.52)	12.29 (7.49)	−5.611 to 8.183	0.396 (33)	.70
K-ARS-in								
Baseline		7 (20)	28 (80)	11.29 (7.65)	9.79 (4.48)	−7.408 to 4.408	−0.682 (33)	.50
Postintervention		7 (20)	28 (80)	6.86 (4.85)	7.00 (3.85)	−3.719 to 4.00	0.084 (33)	.93
K-ARS-hi								
Baseline		7 (20)	28 (80)	10.71 (7.99)	9.18 (5.14)	−7.756 to 4.684	−0.631 (33)	.53
Postintervention		7 (20)	28 (80)	4.14 (4.18)	5.29 (4.35)	−2.349 to 4.634	0.626 (33)	.54
Task 5								
K-ARS-to								
Baseline		9 (25.7)	26 (74.3)	18.89 (11.01)	19.81 (8.92)	−7.047 to 8.885	0.251 (33)	.80
Postintervention		9 (25.7)	26 (74.3)	11.44 (7.21)	12.23 (7.85)	−4.808 to 6.381	0.264 (33)	.79
K-ARS-in								
Baseline		9 (25.7)	26 (74.3)	8.56 (4.82)	10.62 (5.26)	−1.685 to 5.805	1.032 (33)	.31
Postintervention		9 (25.7)	26 (74.3)	6.22 (3.42)	7.23 (4.20)	−1.748 to 3.765	0.648 (33)	.52
K-ARS-hi								
Baseline		9 (25.7)	26 (74.3)	10.33 (7.18)	9.19 (5.25)	−6.245 to 3.963	−0.511 (33)	.61
Postintervention		9 (25.7)	26 (74.3)	5.22 (4.38)	5.00 (4.34)	−3.534 to 3.090	−0.132 (33)	.90

a Negative task difficulty slope group.

b Positive task difficulty slope group.

c K-ARS-to: total score.

d K-ARS-in: inattention score.

e K-ARS-hi: hyperactivity/impulsivity score.

## Discussion

### Principal Findings

This study aimed to investigate whether task difficulty regulation during adaptive DTx training is associated with ADHD symptom improvement and to evaluate whether difficulty-related behavioral measures can serve as clinically meaningful indicators of treatment response. Consistent with our hypothesis, compared with the attainment of higher absolute difficulty levels, stable and individualized regulation of task difficulty was more strongly associated with improvements in ADHD symptoms, particularly hyperactivity and impulsivity. These results suggest that how task demands are dynamically adjusted during training may be a more clinically relevant factor than the overall level of difficulty achieved.

These findings extend prior research on digital interventions for ADHD by shifting the focus from training intensity and duration toward the structure of difficulty adaptation itself. Previous DTx studies have primarily emphasized treatment efficacy, engagement, and repeated exposure, such as in the AKL-T01 randomized trial [11], while adaptive cognitive training frameworks have generally assumed that progressively increasing task difficulty enhances cognitive outcomes [42,43]. These findings challenge this assumption by demonstrating that dynamic regulation of task difficulty may be more critical than the absolute level attained. Excessive or unstable increases in task demands may undermine behavioral stabilization processes, particularly in domains related to hyperactivity and impulsivity. This pattern is consistent with theoretical models of ADHD that emphasize regulatory control deficits and increased intraindividual variability, suggesting that interventions prioritizing stability over escalation may be better aligned with the neurocognitive profile of this population [44].

Task difficulty in DTx is known to modulate cognitive performance in children with ADHD, with excessively high task demands potentially limiting improvements in attention and cognitive control [45,46]. Children with ADHD also exhibit impaired inhibitory control and increased reaction time variability, reflecting instability in regulatory processes during task performance [47,48]. These observations are consistent with our results, which suggest that frequent changes or excessive increases in task demands may impose additional regulatory burdens, require ongoing adjustment of behavioral strategies, and interfere with stable behavioral control. This may be particularly relevant for hyperactivity-related behaviors. Moreover, given that children with ADHD exhibit deficits in self-regulation, including impulse control and behavioral stabilization [49], symptom improvement may be reduced when task demands exceed an individual’s regulatory capacity.

One potential explanation for these findings is that task difficulty functions as a regulatory mechanism that modulates cognitive and behavioral load. High task difficulty or high variance may impose additional demands on inhibitory control and response adaptations, requiring users to continuously recalibrate their behavioral strategies. This process may be particularly challenging for children with ADHD, who typically show deficits in behavioral stability and increased variability in cognitive performance [50]. In addition, fluctuating or overly demanding tasks may increase perceived mental effort and regulatory burden, further interfering with sustained engagement [46]. In this context, stable and appropriately calibrated task difficulty may reduce regulatory burden, thereby facilitating more consistent engagement and behavioral control.

From a clinical and design perspective, these findings have important implications for the development of adaptive DTx systems. First, they suggest that adaptive algorithms should prioritize the stabilization and personalization of task difficulty rather than implementing uniform or progressively increasing difficulty schedules. Second, task difficulty trajectories may serve as potential digital biomarkers that reflect individual differences in regulatory capacity and treatment responsiveness, providing a clinically meaningful metric beyond traditional performance-based measures. This perspective is consistent with emerging approaches in digital phenotyping, which aim to capture dynamic behavioral patterns as indicators of underlying mental health states [51,52]. Finally, by emphasizing the importance of aligning task demands with individual functional capacity, these results support a conceptual shift in the design of digital cognitive interventions from performance maximization to regulation optimization.

In summary, these findings suggest that the clinical effectiveness of adaptive DTx for ADHD depends not on achieving higher levels of task difficulty but on maintaining stable, individualized regulation of task demands over time. Conceptualizing task difficulty as a dynamic regulatory mechanism rather than a performance target provides a novel framework for understanding and optimizing digital interventions for ADHD. This perspective may contribute to the development of more effective, interpretable, and clinically meaningful game-based therapeutic systems.

### Limitations

Two potential limitations must be considered when interpreting the findings. First, the sample size (n=35) was modest, and further validation in larger and more diverse clinical cohorts would strengthen generalizability. Nonetheless, our sample is comparable to or larger than those of other exploratory DTx studies, and the consistent patterns observed across multiple indicators provide preliminary confidence in the reliability of our findings. Second, multiple statistical comparisons were conducted across several task indices and outcome measures. While the results showed meaningful convergence across related analyses, this study was exploratory in nature, and formal correction for multiple comparisons was not applied. Several findings had *P* values close to the conventional significance threshold (approximately 0.04‐0.05), potentially raising the risk of type 1 error, and should be interpreted with caution. Group distributions were limited, which may constrain the precision of performance-symptom correlations, as well as overall statistical power and generalizability. Studies with larger samples would enable more restrictive statistical correction strategies and validation using confirmatory designs. In addition, individual differences in baseline symptom severity and regulatory capacity may moderate responses to adaptive difficulty, which warrants further investigation.

### Future Work

Future research should extend these findings by examining adaptive digital training frameworks that prioritize individualized and stable difficulty regulation across larger and more diverse clinical samples. Such studies should also examine whether adaptive algorithms specifically designed to maintain stable task difficulty lead to greater clinical improvement compared to conventional difficulty escalation approaches. In addition, future studies should systematically investigate how task structure and adaptive control strategies interact to influence symptom-specific outcomes, including hyperactivity and broader behavioral regulation. Such efforts will help refine adaptive algorithms and further enhance the clinical applicability and interpretability of game-based DTx for ADHD.

### Conclusion

This exploratory study presents an innovative perspective by demonstrating that stable and individualized regulation of task difficulty, rather than increases in absolute difficulty levels, is a key determinant of clinical response to adaptive digital cognitive training in children with ADHD. Analysis of detailed in-game behavioral data revealed that improvements in hyperactivity and behavioral regulation were more closely associated with stable, personalized difficulty regulation than with absolute difficulty levels or progressive increases in challenge. These results emphasize the role of task difficulty as a mechanism for modulating cognitive and behavioral load, differentiating this study from previous approaches that assumed a linear relationship between difficulty and symptom improvement. Accordingly, the findings highlight the importance of developing DTx that incorporate personalized difficulty trajectories based on real-time user performance. By advancing both conceptual and methodological frameworks for personalized adaptive DTx, this study contributes to the development of patient-centered interventions with enhanced clinical effectiveness and interpretability in real-world settings.

## Supplementary material

10.2196/83932 Multimedia Appendix 1 Detailed explanation of the digital cognitive training content “Neuro-World” and statistical results for Korean-Child Behavior Checklist.

10.2196/83932 Checklist 1 CONSORT-eHEALTH checklist.

## Abbreviations

ADHD: attention-deficit/hyperactivity disorder
CONSORT-eHEALTH: Consolidated Standards of Reporting Trials of Electronic and Mobile Health Applications and Online Telehealth
*DSM*: *Diagnostic and Statistical Manual of Mental Disorders*
DTx: digital therapeutics
K-ARS: Korean-ADHD Rating Scale
K-CBCL: Korean-Child Behavior Checklist
