# Supplementary material for: Association Between Task Difficulty Regulation in Game-Based Digital Therapeutics and Attention-Deficit/Hyperactivity Disorder Symptoms in Children: Secondary Analysis of a Randomized Controlled Trial
Source: JMIR Serious Games. 2026 Jun 11;14:e83932. doi: 10.2196/83932 (PMC13261165; doi:10.2196/83932)
Supplement: Multimedia Appendix 1 [file games-v14-e83932-s001.docx]

**Task Difficulty Regulation in Game-Based Digital Therapeutics Is Associated with Symptom Improvement in Children with ADHD: Secondary Analysis of an RCT**

**Jun-Su Kim^1,*^, MS | Seung-Jae Kim^1,*^, MS | Gilnam Ryu^1^, MS | Su-Jin Jun^1^, BS | Jin-Yeop Park^1^, BS | Mun-Ju Kim^1^, MS | Sang Sun Han^2^, PhD | So-Hyeon Yoo^2^, PhD | Byeong-II Kim^4^, MS | Hyun-ju Lee^1,2,*^, PhD | Jeong-Heon Song^1,*^, PhD | Hyang-Sook Hoe^1,2,3,*^, PhD**

^1^AI-based Neurodevelopmental Diseases Digital Therapeutics Group, Korea Brain Research Institute (KBRI), 61, Cheomdan-ro, Daegu, 41062, Republic of Korea; ^2^ Department of Neural Development and Disease, Korea Brain Research Institute (KBRI), 61, Cheomdan-ro, Daegu, 41062, Republic of Korea; ^3^ Department of Brain and Cognitive Sciences, Daegu Gyeongbuk Institute of Science & Technology, Daegu 42988, Republic of Korea. ^4^Woorisoft Inc, Daegu 42781, Republic of Korea. ^*^These authors contributed equally to this work.

*** Corresponding authors:**

**Hyun-ju Lee:** Department of Neural Development and Disease, AI-based Neurodevelopmental Diseases Digital Therapeutics Group, Korea Brain Research Institute (KBRI), 61, Cheomdan-ro, Daegu, 41062, Republic of Korea, Tel: 82-53-980-8313; e-mail: hjlee@kbri.re.kr

**Jeong-Heon Song**: AI-based Neurodevelopmental Diseases Digital Therapeutics Group, Korea Brain Research Institute (KBRI), 61, Cheomdan-ro, Daegu, 41062, Republic of Korea, Tel: 82-53-980-5670; e-mail: jhsong@kbri.re.kr

**Hyang-Sook Hoe**: Department of Neural Development and Disease, AI-based Neurodevelopmental Diseases Digital Therapeutics Group, Korea Brain Research Institute (KBRI), 61, Cheomdan-ro, Daegu, 41062, Republic of Korea, Tel: 82-53-980-8310; e-mail: sookhoe72@kbri.re.kr

# Supplementary Methods

## Digital Cognitive Training Content: Neuro-World

The five tasks were **Task 1:** recognizing common features among animal figures under continuously changing visual conditions; **Task 2:** rearranging animal sequences to match target patterns; **Task 3:** memorizing and recalling the positions of matching pairs of animals; **Task 4:** coordinating dual spaceship control to collect targets and avoid obstacles; and **Task 5:** independently controlling left/right spaceships for selective target collection and obstacle avoidance.

| Category | Neuro-World Content |
| --- | --- |
| **Task 1** | 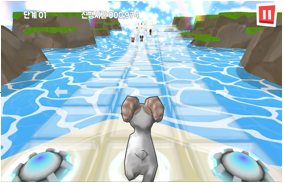 |
|  | - Content in which the user identifies the animal they control and matches it with the same animal among three approaching animals. - Continuous sustained attention is required despite changes in running speed and viewpoint caused by rail height variation; the user must remember the shape of the animal even as the rail height and position change and determine whether it matches the upcoming target to complete the match. - The viewpoint shifts according to rail height and lateral movement, and both rail elevation and running speed increase with content difficulty; thus, the user must progress through the content while adapting to the accelerating speed. |
| **Task 2** | 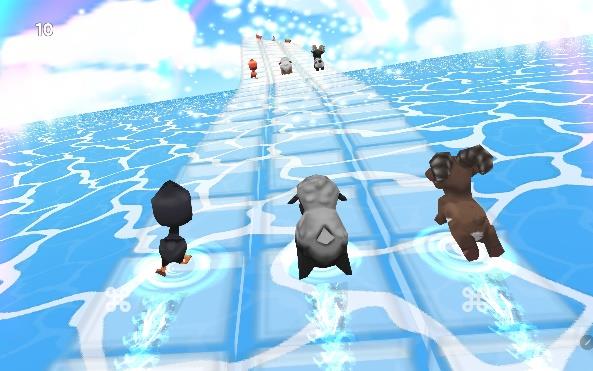 |
|  | - Content in which the user checks the types and order of three animals displayed at the bottom of the screen and matches them to the order of the approaching animals. - The user observes the order of the three animals in front and rearranges them to match the order of the three approaching animals. Pressing the left button swaps the left and center animals, while pressing the right button swaps the right and center animals. - The viewpoint changes depending on rail height and lateral movement, and running speed increase with content difficulty; thus, the user must keep pace with the increasing speed. |
| **Task 3** | 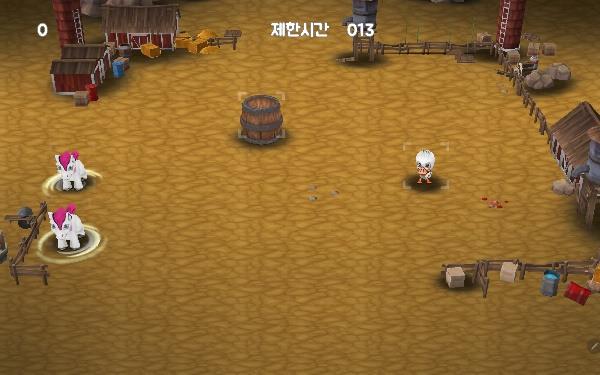 |
|  | - Content in which the user memorizes pairs of animals displayed on the screen and later finds the matching pairs hidden behind covered logs. - The user memorizes animal pairs shown on the screen and, after a set time when all animals are hidden inside logs, must find them. If a pair is not matched correctly, the animals are hidden again, requiring the user to remember previously revealed animals even after incorrect attempts. - As difficulty increases, the number of animal pairs gradually rises, increasing overall complexity. |
| **Task 4** | 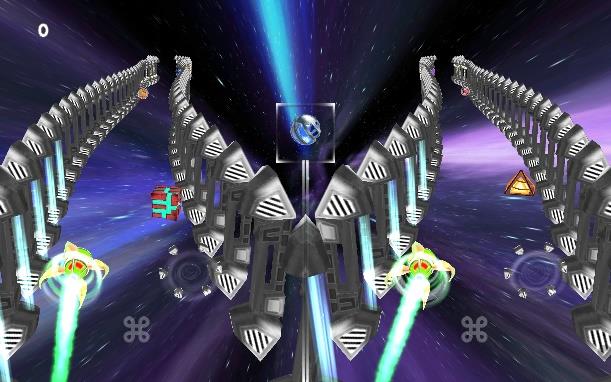 |
|  | - Content in which the user identifies the positions of approaching obstacles and compares them with the positions of two spacecraft. - The user must focus on both spacecraft on the left and right, controlling the left rail with the left hand and the right rail with the right hand. By observing the paths of approaching obstacles, the user must change the spacecrafts’ paths to prevent collisions. If a model displayed on both sides of the screen appears among the obstacles, the user must collect it. - To account for cases where the paths of approaching obstacles are not visible due to rail curvature, the user must identify obstacle positions in advance. |
| **Task 5** | 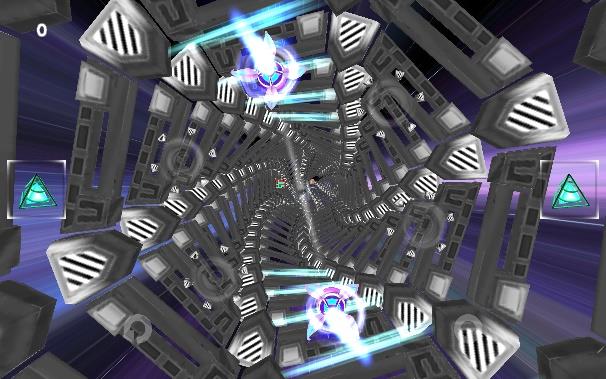 |
|  | - Content in which the user identifies the path that their spacecrafts will take and the paths of approaching obstacles inside a rotating hexagonal rail. - The user observes the paths of two paired spacecraft and approaching obstacles and adjusts the spacecrafts’ paths so that neither spacecraft collides with obstacles. If a model displayed on both sides of the screen appears among the obstacles, the user must collect it. Touching the left side moves the spacecraft counterclockwise, while touching the right side moves it clockwise. - As the user’s viewpoint tilts along with the inclination of the rotating hexagonal rail, sustained concentration is required despite continuous viewpoint changes, and the user must assess both the approaching obstacles and the spacecrafts’ positions. |

**Supplementary Table 1**. Multiple regression analysis of average task difficulty and K-CBCL scores in DTx invention participants

| Outcome Measure | Covariate (Baseline) | Content Task | Coefficient (β) | 95% CI | t-statistic | p-value |
| --- | --- | --- | --- | --- | --- | --- |
| K-CBCL-to  (Post) | K-CBCL-to  (Baseline) | Task 1 | -0.147 | (-0.850, 0.557) | -0.425 | 0.674 |
|  |  | Task 2 | -0.231 | (-0.907, 0.444) | -0.697 | 0.491 |
|  |  | Task 3 | 0.115 | (-3.987, 4.218) | 0.057 | 0.955 |
|  |  | Task 4 | -0.055 | (-0.740, 0.630) | -0.164 | 0.871 |
|  |  | Task 5 | -0.062 | (-0.811, 0.688) | -0.168 | 0.868 |
| K-CBCL-in  (Post) | K-CBCL-in  (Baseline) | Task 1 | -0.064 | (-0.951, 0.822) | -0.148 | 0.883 |
|  |  | Task 2 | -0.179 | (-1.026, 0.669) | -0.429 | 0.671 |
|  |  | Task 3 | 0.023 | (-5.120, 5.166) | 0.009 | 0.993 |
|  |  | Task 4 | -0.422 | (-1.261, 0.418) | -1.023 | 0.314 |
|  |  | Task 5 | 0.065 | (-0.872, 1.002) | 0.141 | 0.888 |
| K-CBCL-ex  (Post) | K-CBCL-ex  (Baseline) | Task 1 | -0.187 | (-0.952, 0.579) | -0.496 | 0.623 |
|  |  | Task 2 | -0.299 | (-1.032, 0.435) | -0.829 | 0.413 |
|  |  | Task 3 | 0.084 | (-4.380, 4.548) | 0.038 | 0.970 |
|  |  | Task 4 | -0.161 | (-0.907, 0.585) | -0.44 | 0.663 |
|  |  | Task 5 | -0.369 | (-1.183, 0.444) | -0.925 | 0.362 |

K-CBCL-to: total score, K-CBCL-in: internalize score, K-CBCL-ex: externalizing score

**Supplementary Table 2**. Multiple regression analysis of task difficulty variability and K-CBCL scores in DTx invention participants

| Outcome Measure | Covariate (Baseline) | Content Task | Coefficient (β) | 95% CI | t-statistic | p-value |
| --- | --- | --- | --- | --- | --- | --- |
| K-CBCL-to  (Post) | K-CBCL-to  (Baseline) | Task 1 | -0.325 | (-2.664, 2.014) | -0.283 | 0.779 |
|  |  | Task 2 | -0.539 | (-2.435, 1.357) | -0.579 | 0.566 |
|  |  | Task 3 | 0.716 | (-3.887, 5.318) | 0.317 | 0.754 |
|  |  | Task 4 | -0.262 | (-1.786, 1.262) | -0.35 | 0.728 |
|  |  | Task 5 | -0.063 | (-1.626, 1.501) | -0.082 | 0.935 |
| K-CBCL-in  (Post) | K-CBCL-in  (Baseline) | Task 1 | 0.35 | (-2.537, 3.237) | 0.247 | 0.806 |
|  |  | Task 2 | -0.179 | (-2.546, 2.189) | -0.154 | 0.879 |
|  |  | Task 3 | 1.543 | (-4.164, 7.250) | 0.551 | 0.586 |
|  |  | Task 4 | -1.175 | (-3.013, 0.663) | -1.302 | 0.202 |
|  |  | Task 5 | 0.025 | (-1.935, 1.984) | 0.026 | 0.980 |
| K-CBCL-ex  (Post) | K-CBCL-ex  (Baseline) | Task 1 | -1.909 | (-4.360, 0.541) | -1.587 | 0.122 |
|  |  | Task 2 | -1.466 | (-3.472, 0.541) | -1.488 | 0.147 |
|  |  | Task 3 | 0.401 | (-4.610, 5.412) | 0.163 | 0.872 |
|  |  | Task 4 | -0.369 | (-2.026, 1.289) | -0.453 | 0.654 |
|  |  | Task 5 | -0.618 | (-2.328, 1.092) | -0.737 | 0.467 |

K-CBCL-to: total score, K-CBCL-in: internalize score, K-CBCL-ex: externalizing score

**Supplementary Tables 3**. Independent-samples t-test results comparing baseline and post-intervention K-CBCL outcomes between positive and negative task difficulty slope groups

| Performance feature | Psychological variable | Session | N (A^a^) | N (B^b^) | Mean (A^a^) | Mean (B^b^) | 95% CI | t-statistic | p-value |
| --- | --- | --- | --- | --- | --- | --- | --- | --- | --- |
| Task 1 | K-CBCL-to | Baseline | 19 | 16 | 65.89 | 69.13 | (-4.031, 10.492) | 0.908 | 0.371 |
|  |  | Post | 19 | 16 | 56.32 | 58.44 | (-4.404, 8.648) | 0.651 | 0.520 |
|  | K-CBCL-in | Baseline | 19 | 16 | 62.11 | 65.19 | (-4.080, 10.245) | 0.880 | 0.385 |
|  |  | Post | 19 | 16 | 53.74 | 55.63 | (-4.997, 8.773) | 0.555 | 0.582 |
|  | K-CBCL-ex | Baseline | 19 | 16 | 63.74 | 65.44 | (-6.069, 9.470) | 0.433 | 0.668 |
|  |  | Post | 19 | 16 | 55.58 | 56.88 | (-4.725, 7.318) | 0.432 | 0.669 |
| Task 2 | K-CBCL-to | Baseline | 10 | 25 | 66.10 | 67.88 | (-5.382, 8.942) | 0.449 | 0.656 |
|  |  | Post | 10 | 25 | 57.00 | 57.40 | (-6.193, 6.993) | 0.111 | 0.913 |
|  | K-CBCL-in | Baseline | 10 | 25 | 63.40 | 63.56 | (-5.111, 5.431) | 0.041 | 0.968 |
|  |  | Post | 10 | 25 | 54.70 | 54.56 | (-6.750, 6.470) | -0.037 | 0.971 |
|  | K-CBCL-ex | Baseline | 10 | 25 | 63.10 | 65.08 | (-6.585, 10.545) | 0.457 | 0.651 |
|  |  | Post | 10 | 25 | 55.10 | 56.60 | (-4.535, 7.535) | 0.453 | 0.653 |
| Task 3 | K-CBCL-to | Baseline | 19 | 16 | 67.37 | 67.38 | (-7.270, 7.283) | 0.002 | 0.999 |
|  |  | Post | 19 | 16 | 57.79 | 56.69 | (-7.784, 5.580) | -0.336 | 0.739 |
|  | K-CBCL-in | Baseline | 19 | 16 | 62.74 | 64.44 | (-5.364, 8.766) | 0.482 | 0.633 |
|  |  | Post | 19 | 16 | 54.89 | 54.25 | (-7.640, 6.351) | -0.189 | 0.851 |
|  | K-CBCL-ex | Baseline | 19 | 16 | 65.84 | 62.94 | (-10.616, 4.807) | -0.743 | 0.463 |
|  |  | Post | 19 | 16 | 57.16 | 55.00 | (-8.138, 3.822) | -0.723 | 0.475 |
| Task 4 | K-CBCL-to | Baseline | 7 | 28 | 69.00 | 66.96 | (-10.388, 6.317) | -0.455 | 0.652 |
|  |  | Post | 7 | 28 | 59.14 | 56.82 | (-9.795, 5.152) | -0.571 | 0.572 |
|  | K-CBCL-in | Baseline | 7 | 28 | 61.14 | 64.11 | (-6.254, 12.183) | 0.676 | 0.504 |
|  |  | Post | 7 | 28 | 54.43 | 54.64 | (-7.975, 8.404) | 0.050 | 0.960 |
|  | K-CBCL-ex | Baseline | 7 | 28 | 70.14 | 63.11 | (-18.496, 4.425) | -1.480 | 0.148 |
|  |  | Post | 7 | 28 | 58.29 | 55.64 | (-10.146, 4.861) | -0.711 | 0.482 |
| Task 5 | K-CBCL-to | Baseline | 9 | 26 | 66.00 | 67.85 | (-3.961, 7.654) | 0.451 | 0.655 |
|  |  | Post | 9 | 26 | 58.44 | 56.88 | (-7.175, 4.055) | -0.418 | 0.678 |
|  | K-CBCL-in | Baseline | 9 | 26 | 60.00 | 64.73 | (-3.147, 12.608) | 1.196 | 0.240 |
|  |  | Post | 9 | 26 | 54.33 | 54.69 | (-6.406, 7.124) | 0.092 | 0.927 |
|  | K-CBCL-ex | Baseline | 9 | 26 | 66.00 | 64.00 | (-10.131, 6.131) | -0.447 | 0.658 |
|  |  | Post | 9 | 26 | 58.11 | 55.50 | (-8.804, 3.582) | -0.768 | 0.448 |

a: Negative task difficulty slope group, b: Positive task difficulty slope group
K-CBCL-to: total score, K-CBCL-in: internalize score, K-CBCL-ex: externalizing score
